# Identification of serum metabolome signatures associated with retinal and renal complications of type 2 diabetes

**DOI:** 10.1038/s43856-022-00231-3

**Published:** 2023-01-09

**Authors:** Yoshihiko Tomofuji, Ken Suzuki, Toshihiro Kishikawa, Nobuhiro Shojima, Jun Hosoe, Kyoko Inagaki, Sunao Matsubayashi, Hisamitsu Ishihara, Hirotaka Watada, Yasushi Ishigaki, The BioBank Japan Project, Yuji Yamanashi, Yoichi Furukawa, Takayuki Morisaki, Yoichiro Kamatani, Kaori Muto, Akiko Nagai, Wataru Obara, Ken Yamaji, Kazuhisa Takahashi, Satoshi Asai, Yasuo Takahashi, Takao Suzuki, Nobuaki Sinozaki, Hiroki Yamaguchi, Shiro Minami, Shigeo Murayama, Kozo Yoshimori, Satoshi Nagayama, Daisuke Obata, Masahiko Higashiyama, Akihide Masumoto, Yukihiro Koretsune, Hidenori Inohara, Yoshinori Murakami, Koichi Matsuda, Yukinori Okada, Toshimasa Yamauchi, Takashi Kadowaki

**Affiliations:** 1 Department of Statistical Genetics, Osaka University Graduate School of Medicine, Osaka, Japan; 2 Integrated Frontier Research for Medical Science Division, Institute for Open and Transdisciplinary Research Initiatives, Osaka University, Osaka, Japan; 3 Department of Diabetes and Metabolic Diseases, Graduate School of Medicine, The University of Tokyo, Tokyo, Japan; 4 Centre for Genetics and Genomics Versus Arthritis, Centre for Musculoskeletal Research, Division of Musculoskeletal and Dermatological Sciences, University of Manchester, Manchester, England UK; 5 Department of Otorhinolaryngology-Head and Neck Surgery, Osaka University Graduate School of Medicine, Osaka, Japan; 6 Department of Head and Neck Surgery, Aichi Cancer Center Hospital, Aichi, Japan; 7 Division of Diabetes, Endocrinology, and Metabolism, Department of Medicine, Nippon Medical School, Tokyo, Japan; 8 Fukuoka Tokushukai Hospital, Fukuoka, Japan; 9 Division of Diabetes and Metabolism, Nihon University School of Medicine, Tokyo, Japan; 10 Department of Metabolism & Endocrinology, Juntendo University Graduate School of Medicine, Tokyo, Japan; 11 Division of Diabetes, Metabolism and Endocrinology, Iwate Medical University, Iwate, Japan; 12 Division of Molecular Pathology, Institute of Medical Science, The University of Tokyo, Tokyo, Japan; 13 Department of Computational Biology and Medical Sciences, Graduate school of Frontier Sciences, The University of Tokyo, Tokyo, Japan; 14 Laboratory of Statistical Immunology, Immunology Frontier Research Center (WPI-IFReC), Osaka University, Osaka, Japan; 15 Laboratory for Systems Genetics, RIKEN Center for Integrative Medical Sciences, Kanagawa, Japan; 16 Department of Genome Informatics, Graduate School of Medicine, the University of Tokyo, Tokyo, Japan; 17 AMED-CREST, Japan Agency for Medical Research and Development, Tokyo, Japan; 18 Toranomon Hospital, Tokyo, Japan; 19 Department of Prevention of Diabetes and Life-style Related Diseases, The University of Tokyo, Tokyo, Japan; 20 Division of Genetics, The Institute of Medical Science, The University of Tokyo, Tokyo, Japan; 21 Division of Clinical Genome Research, Institute of Medical Science, The University of Tokyo, Tokyo, Japan; 22 Division of Molecular Pathology, IMSUT Hospital Department of Internal Medicine, Institute of Medical Science, The University of Tokyo, Tokyo, Japan; 23 Laboratory of Complex Trait Genomics, Graduate School of Frontier Sciences, The University of Tokyo, Tokyo, Japan; 24 Laboratory of Clinical Genome Sequencing, Graduate School of Frontier Sciences, The University of Tokyo, Tokyo, Japan; 25 Department of Public Policy, Institute of Medical Science, The University of Tokyo, Tokyo, Japan; 26 Department of Urology, Iwate Medical University, Iwate, Japan; 27 Department of Internal Medicine and Rheumatology, Juntendo University Graduate School of Medicine, Tokyo, Japan; 28 Department of Respiratory Medicine, Juntendo University Graduate School of Medicine, Tokyo, Japan; 29 Division of Pharmacology, Department of Biomedical Science, Nihon University School of Medicine, Tokyo, Japan; 30 Division of Genomic Epidemiology and Clinical Trials, Clinical Trials Research Center, Nihon University. School of Medicine, Tokyo, Japan; 31 Tokushukai Group, Tokyo, Japan; 32 Departmentof Hematology, Nippon Medical School, Tokyo, Japan; 33 Department of Bioregulation, Nippon Medical School, Kawasaki, Japan; 34 Tokyo Metropolitan Geriatric Hospital and Institute of Gerontology, Tokyo, Japan; 35 Fukujuji Hospital, Japan Anti-Tuberculosis Association, Tokyo, Japan; 36 The Cancer Institute Hospital of the Japanese Foundation for Cancer Research, Tokyo, Japan; 37 Center for Clinical Research and Advanced Medicine, Shiga University of Medical Science, Shiga, Japan; 38 Department of General Thoracic Surgery, Osaka International Cancer Institute, Osaka, Japan; 39 IIZUKA HOSPITAL, Fukuoka, Japan; 40 National Hospital Organization Osaka National Hospital, Osaka, Japan

**Keywords:** Metabolomics, Diagnostic markers, Diabetes complications

## Abstract

**Background:**

Type 2 diabetes is a common disease around the world and its major complications are diabetic retinopathy (DR) and diabetic kidney disease (DKD). Persons with type 2 diabetes with complications, especially who have both DR and DKD, have poorer prognoses than those without complications. Therefore, prevention and early identification of the complications of type 2 diabetes are necessary to improve the prognosis of persons with type 2 diabetes. The aim of this study is to identify factors associated with the development of multiple complications of type 2 diabetes.

**Methods:**

We profiled serum metabolites of persons with type 2 diabetes with both DR and DKD (*N* = 141) and without complications (*N* = 159) using a comprehensive non-targeted metabolomics approach with mass spectrometry. Based on the serum metabolite profiles, case–control comparisons and metabolite set enrichment analysis (MSEA) were performed.

**Results:**

Here we show that five metabolites (cyclohexylamine, *P* = 4.5 × 10^−6^; 1,2-distearoyl-glycero-3-phosphocholine, *P* = 7.3 × 10^−6^; piperidine, *P* = 4.8 × 10^−4^; *N*-acetylneuraminic acid, *P* = 5.1 × 10^−4^; stearoyl ethanolamide, *P* = 6.8 × 10^−4^) are significantly increased in those with the complications. MSEA identifies fatty acid biosynthesis as the type 2 diabetes complications-associated biological pathway (*P* = 0.0020).

**Conclusions:**

Our metabolome analysis identifies the serum metabolite features of the persons with type 2 diabetes with multiple complications, which could potentially be used as biomarkers.

## Plain language summary

In the management of type 2 diabetes, prevention and early identification of diabetes complications are important. In particular, people with type 2 diabetes with diabetic retinopathy (DR), affecting the eye, and diabetic kidney disease (DKD), have poorer outcomes than those without complications and need early intervention. Here, we comprehensively profiled blood metabolites, or breakdown products of the biological processes occurring in the body, of people with type 2 diabetes with both DR and DKD and those without complications. We found that five metabolites were significantly increased in those with complications, and we identified a specific metabolic pathway associated with having complications. Our analysis identified the blood metabolite features of people with type 2 diabetes with multiple complications, which could potentially be used as markers in the future.

## Abstract (web summary)

Tomofuji et al. perform non-targeted metabolomics on serum samples from people with type 2 diabetes with or without complications. The authors identify metabolites associated with retinal and renal complications.

## Introduction

Type 2 diabetes (T2D), which is caused by multifactorial pathogenesis, is one of the most common diseases around the world. 6.3% of the world’s population is estimated to be affected by T2D, and the prevalence of T2D is still growing^1^. Although the symptoms of T2D are often mild in its early stage, long-term complications such as micro- and macro-vascular diseases are often critical. Therefore, one of the major goals of clinical care for T2D is preventing critical complications by multifactorial intervention strategy to strictly control glucose, blood pressure, and other risk factors^2^.

Diabetic retinopathy (DR) and diabetic kidney disease (DKD) are major long-term complications of diabetes (develops in 20% and 30–40% of persons with diabetes, respectively)^3,4^. DR is one of the major causes of vision loss in the world (0.86 million cases in those aged 50 years and older in 2020)^5^. DKD is the leading cause of chronic kidney disease (CKD), and ~50% of the end-stage renal disease was due to DKD in developed countries^6^. DKD is a major risk factor for mortality in persons with T2D^7^. In addition, persons with T2D with both DR and DKD have a much higher risk of mortality than those with only DR or DKD (compared with those with no DR or DKD, the hazard ratios for all-cause mortality were 2.76, 1.89, 1.38, respectively for those with both DR and DKD, only DKD, and only DR)^8^. Although several risk factors for the complications of T2D, such as duration of T2D, poor glycemic control, and hypertension were identified, some persons with T2D without tightly controlled blood glucose and blood pressure sometimes fail to develop the complications of T2D. Therefore, it is necessary to identify additional biomarkers which are associated with the complications of T2D for the improvement of the care for persons with T2D.

High-throughput metabolomics, which can simultaneously profile multiple metabolites in samples, emerged as a useful technology for identifying changes in metabolic signatures in disease conditions. The two most common techniques used in high-throughput metabolomics are nuclear magnetic resonance (NMR) and mass spectrometry (MS), and the latter achieve comparatively high sensitivity and a large number of detectable metabolites. Many studies have already been conducted and identified the association between T2D and several metabolites such as branched-chain amino acid, aromatic amino acid, glycine, and 2-hydroxybutyric acid^9^. The association between metabolic profiles and complications of T2D was also investigated in several studies. It has been reported that blood metabolites related to amino acid metabolism, nucleic acid metabolism, glycolysis, and fatty acid metabolism were associated with DR^10–13^. As for DKD, associations with various blood metabolites related to the tricarboxylic acid cycle, amino acid metabolism, uric acid metabolism, nucleic acid metabolism, and fatty acid metabolism have been reported^14^. However, the number of participants and evaluated metabolites were often small in these studies, which potentially resulted in less consistent findings. In addition, metabolic signatures of persons with T2D with both DR and DKD were not fully evaluated, even though they have the highest risk of mortality among persons with T2D.

Here, we revealed the serum metabolite signatures of persons with T2D with both DR and DKD with a comprehensive non-targeted metabolomics approach combining capillary electrophoresis time-of-flight mass spectrometry (CE-TOFMS) and liquid chromatography TOFMS (LC-TOFMS). We compared the abundance of the 364 serum metabolites between the persons with T2D with both DR and DKD (*N* = 141) and those without either DR or DKD (*N* = 159). We performed the sub-analyses, including additional covariates, to evaluate the robustness of the association between the complications and the metabolites. Additionally, we performed metabolite set enrichment analysis (MSEA) to identify the biological pathways related to the complications of T2D. Through these analyses, we reveal that several serum metabolites, including *N*-acetylneuraminic acid, and fatty acid biosynthesis-related pathways are associated with complications of type 2 diabetes.

## Methods

### Subject participation

We examined 141 persons with T2D with both DR and DKD as cases and 159 persons with T2D without either DR or DKD as controls. All the subjects were registered in the BioBank Japan (BBJ) project^15^ and the complications of diabetes were defined based on the medical records in which diagnosis was made by physicians at the participating hospitals. The persons with T2D with the complications in our cohort had pre-proliferative or proliferative diabetic retinopathy and mild diabetic kidney disease with micro- or macro-albuminuria. The characteristics of the cohort are described in Table 1. All the participants provided written informed consent following the protocols approved by individual institutional ethical committees before enrollment. The study protocol was approved by the ethics committees at The University of Tokyo and Osaka University (reference number: 734-15).

**Table 1:** Subject characteristics.

	T2D without complications(control)	T2D with complications(DR + DKD)
Number	159	141
Male/Female	91 / 68	83 / 58
Age (years)	68.2 ± 9.7	64.9 ± 8.9
BMI (kg/m^2^)	23.8 ± 3.7 (12)^a^	23.7 ± 3.7 (6)^a^
Systolic BP (mmHg)	134.1 ± 17.3 (27)^a^	135.8 ± 18.3 (10)^a^
Diastolic BP (mmHg)	75.6 ± 10.8 (27)^a^	75.9 ± 10.9 (10)^a^
Mean BP (mmHg)	95.1 ± 11.6 (27)^a^	95.9 ± 12.0 (10)^a^
HbA1c (%)	7.0 ± 1.3 (47)^a^	7.3 ± 1.3 (32)^a^
sCre (mg/dL)	0.82 ± 0.32 (2)^a^	0.93 ± 0.34 (2)^a^
eGFR (mL/min/1.73m^2^)	70.4 ± 20.1 (2)^a^	64.4 ± 23.2 (2)^a^
Duration (years)	16.6 ± 6.2	12.5 ± 8.5

*BMI* body mass index, *BP* blood pressure, *DKD* diabetic kidney disease, *DR* diabetic retinopathy, *eGFR* estimated glomerular filtration rate, *HbA1c* hemoglobin A1c, *sCre* serum creatinine, *T2D* type 2 diabetes.

^a^Numbers of participants with missing values are indicated in parentheses.

### Sample collection and metabolome profiling

Serum metabolite profiling was performed following the methods used in ref. ^16^. In detail, serum samples from the participants were collected at collaborating facilities. Metabolite extraction and metabolome analysis were conducted at Human Metabolome Technologies (HMT), Japan.

For CE-TOFMS analysis, 50 μl of serum was added to 450 μl of methanol containing internal standards (H3304-1002, HMT) at 0 °C to inactivate enzymes. The internal standards were L-methionine sulfone and D-camphor-10-sulfonic acid for cationic mode and anionic mode, respectively. The extract solution was thoroughly mixed with 500 μl of chloroform and 200 μl of Milli-Q water and centrifuged at 2300 × *g* and 4 °C for 5 min. The 350 μl of the upper aqueous layer was centrifugally filtered through a Millipore 5-kDa cutoff filter to remove proteins. The filtrate was centrifugally concentrated and resuspended in 50 μl of Milli-Q water for CE-MS analysis.

For LC-TOFMS analysis, 500 μl of serum was added to 1500 μl of 1% formic acid/acetonitrile containing internal standard solution (Solution ID: H3304-1002, HMT) at 0 °C to inactivate enzymes. D-camphor-10-sulfonic acid was used for the internal standard in both the positive and negative modes. The solution was thoroughly mixed and centrifuged at 2300 × *g* and 4 °C for 5 min. The supernatant was filtrated by using Hybrid SPE phospholipid (55261-U, Supelco, Bellefonte, PA, USA) to remove phospholipids. The filtrate was desiccated and dissolved with 100 μl of iso-propanol/Milli-Q for LC-MS analysis.

Metabolome analysis was conducted with CE-TOFMS and LC-TOFMS for ionic and nonionic metabolites, respectively. CE-TOFMS analysis was carried out using an Agilent CE system equipped with an Agilent 6210 TOFMS, Agilent 1100 isocratic HPLC pump, Agilent G1603A CE-MS adapter kit, and Agilent G1607A CE-ESI-MS sprayer kit (Agilent Technologies, Santa Clara, CA, USA). The systems were controlled by Agilent G2201AA ChemStation software version B.03.01 for CE (Agilent Technologies) and connected by a fused silica capillary (50 μm i.d. × 80 cm total length) with electrophoresis buffer (H3301-1001 and I3302-1023 for cation and anion analyses, respectively, HMT) as the electrolyte. The spectrometer was scanned from m/z 50 to 1000. LC-TOFMS analysis was carried out using an Agilent LC System (Agilent 1200 series RRLC system SL) equipped with an Agilent 6230 TOFMS (Agilent Technologies). The systems were controlled by Agilent G2201AA ChemStation software version B.03.01 (Agilent Technologies) equipped with an ODS column (2 × 50 mm, 2 μm). The equilibration time was 7.5 min. For every ten samples, the sensitivity of the analysis was confirmed by measuring the D-camphor-10-sulfonic acid solution.

Peaks were extracted using MasterHands, automatic integration software (Keio University, Tsuruoka, Yamagata, Japan) to obtain peak information including *m*/*z*, peak area, and migration time for CE-TOFMS measurement (MT) or retention time for LC-TOFMS measurement (RT). Signal peaks corresponding to isotopomers, adduct ions, and other product ions of known metabolites were excluded. The remaining peaks were annotated according to the HMT metabolite database based on their m/z values with the MTs and RTs determined by TOFMS. Areas of the annotated peaks were normalized based on the levels of the internal standard for each modality (CE-TOFMS, L-methionine sulfone and D-camphor-10-sulfonic acid for cationic mode and anionic mode, respectively; LC-TOFMS, D-camphor-10-sulfonic acid) and sample amounts to obtain relative levels of each metabolite.

### Association tests between metabolites and complications of T2D

Among the 533 metabolites which were detected, 364 metabolites that were detected in ≥20% of the samples were retained for further analysis. The abundance of the metabolite was normalized by log transformation. We added the pseudo-counts (half of the minimum non-zero value) to the zeros before log transformation. Linear regression with the following formula was performed with the lm() function in the R to obtain the covariate-adjusted metabolite abundances; metabolite abundance ~ age + age^2^ + sex + age × sex + duration of type 2 diabetes + top five principal components (Supplementary Fig. 1a). Then, the residuals of the linear regression (i.e., covariate-adjusted metabolite abundance) were regressed to the presence of the complications with the logistics regression model as implemented in the glm() function in the R. False discovery ratio (FDR) was calculated by Benjamini-Hochberg procedure. In the sub-analyses, hemoglobin A1c (HbA1c), mean blood pressure (mBP), body mass index (BMI), serum creatinine (sCre), and estimated glomerular filtration rate (eGFR) were included as covariates (Supplementary Fig. 1b,c). Since this information could not be obtained for some of the subjects (Table 1), we performed sub-analyses only with those who did not have missing values. We performed Wilcoxon rank sum tests based on the raw abundances of the metabolites with wilcox_test() function in the R-coin package.

### Biological pathway enrichment analysis of the serum metabolite

We performed a MSEA using the R package fgsea (version 1.16.0). Forty-four metabolite sets that contained more than five metabolites were included in the enrichment analysis. For case–control pathway association tests, metabolites annotated by the Kyoto Encyclopedia of Genes and Genomes (KEGG) database were ranked based on their *z* value in the case–control metabolite association tests. The KEGG metabolite sets were defined according to the KEGG pathway. False discovery ratio (FDR) was calculated by the Benjamini-Hochberg procedure.

### Statistics and reproducibility

In total, 300 subjects were included in this study (*N*_DR + DKD_ = 141 and *N*_control_ = 159). The association between the metabolite abundances and the presence of complications was evaluated by linear regression and logistic regression as indicated above. We also confirmed the differences in the metabolite abundances between the two groups, namely those with and without complications, by Wilcoxon rank sum tests. For the MSEA, statistical significance was evaluated based on the permutation procedure as default implemented in the R package fgsea. All of the above analyses were conducted with R (version 4.0.1). All samples were used once. Multiple testing was corrected with the FDR method (Benjamini-Hochberg procedure).

## Results

**Fig. 1:**
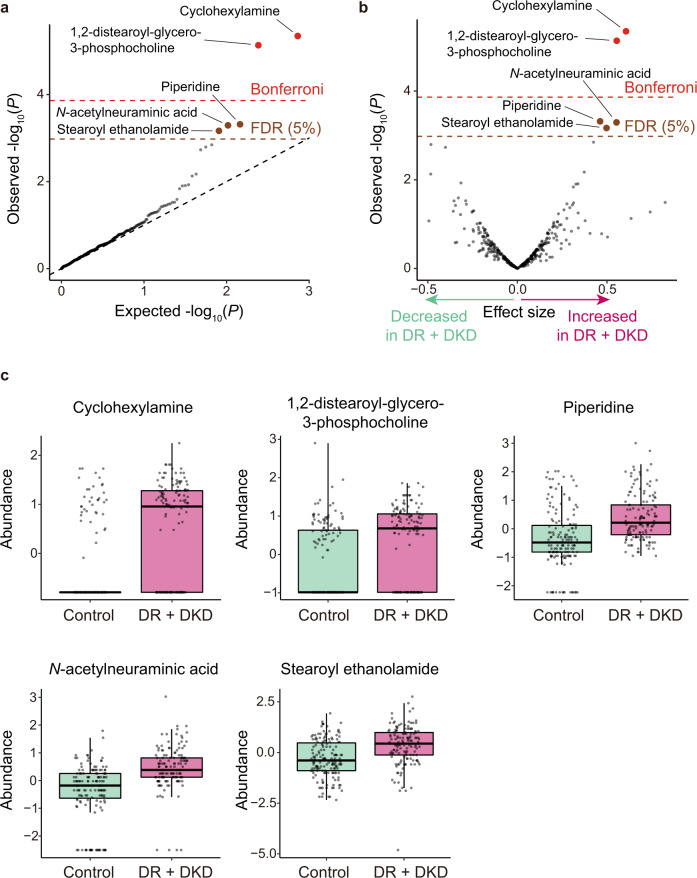
Results of the case–control association tests for metabolites. **a** A quantile–quantile plot of the *p* values in the logistic regression analysis. The *x*-axis indicates log-transformed expected *p* values. The *y*-axis indicates log-transformed observed *p* values. The diagonal dashed line represents *y* = *x*, which corresponds to the null hypothesis. The horizontal red line indicates the Bonferroni-corrected threshold (*α* = 0.05), and the brown line indicates the FDR threshold (FDR = 0.05) calculated by the Benjamini-Hochberg procedure. Metabolites with *p* values less than the Bonferroni thresholds are plotted as red dots, metabolites with *p* values less than the FDR thresholds are plotted as brown dots, and other metabolites are plotted as black dots. **b** A volcano plot. The *x*-axis indicates effect sizes in logistic regression. The *y*-axis, horizontal lines, and dot colors are the same as in **a**. **c** Boxplots for the five metabolites which were significantly associated with the complications of T2D. Boxplots indicate the median values (center lines) and IQR (box edges), with the whiskers extending to the most extreme points within the range between (lower quantile − [1.5 × IQR]) and (upper quantile + [1.5 × IQR]). The number of samples used for the analysis is *N*_DR + DKD_ = 141 and *N*_control_ = 159. DKD, diabetic kidney disease; DR diabetic retinopathy; FDR, false discovery ratio; IQR, interquartile ranges; T2D, type 2 diabetes.

We performed non-targeted metabolomic profiling for 141 persons with T2D with both retinal and renal complications (DR + DKD), and 159 persons with T2D without either DR or DKD (control; Table 1) in BBJ cohort. We tested the association between the complications of T2D and 364 metabolites with adjustment of age, age^2^, sex, age × sex, duration of T2D, and top 5 principal components as previously described^16^. Significant associations were identified for the five metabolites (cyclohexylamine, effect size = 0.606, standard error (SE) = 0.132, and *P* = 4.5 × 10^−6^; 1,2-distearoyl-glycero-3-phosphocholine, effect size = 0.554, SE = 0.124, and *P* = 7.3 × 10^−6^; piperidine, effect size = 0.462, SE = 0.132, and *P* = 4.8 × 10^−4^; *N*-acetylneuraminic acid, effect size = 0.553, SE = 0.159, and *P* = 5.1 × 10^−4^; stearoyl ethanolamide, effect size = 0.497, SE = 0.146, and *P* = 6.8 × 10^−4^; FDR < 0.05; Fig. 1a, Table 2). According to the volcano plot, all of these metabolites increased in the persons with T2D with both retinal and renal complications (DR + DKD) (Fig. 1b). Moreover, boxplots confirmed that all of these metabolites increased in the persons with T2D with DR + DKD compared to those without either DR or DKD (control) (Fig. 1c). We evaluated the relationship between the measurement orders and raw metabolite abundances and found no consistent gradual changes or successive outliers which suggested an intra-batch effect (Supplementary Fig. 2). In addition, we successfully confirmed these associations in a nonparametric statistical method (Wilcoxon rank sum test, *P* < 7.1 × 10^−8^; Supplementary Table 1), suggesting that these associations were robust to the choices of the statistical methods. Among the five metabolites which had significant associations with the complications of T2D, *N*-acetylneuraminic acid was a major form of sialic acid in humans. Sialic acid is reported to be associated with DKD^17^ and DR^18^ in independent studies, supporting the robustness of the associations identified by our analysis.

**Table 2:** Significant associations between the complications of T2D and the metabolites.

Metabolite name	Effect size	SE	*P*	*q*
Cyclohexylamine	0.606	0.132	4.5 × 10^−6^	0.0013
1,2-distearoyl-glycero-3-phosphocholine	0.554	0.124	7.3 × 10^−6^	0.0013
Piperidine	0.462	0.132	4.8 × 10^−4^	0.046
N-acetylneuraminic acid	0.553	0.159	5.1 × 10^−4^	0.046
Stearoyl ethanolamide	0.497	0.146	6.8 × 10^−4^	0.050

*SE* standard error, *T2D* type 2 diabetes.

**Fig. 2:**
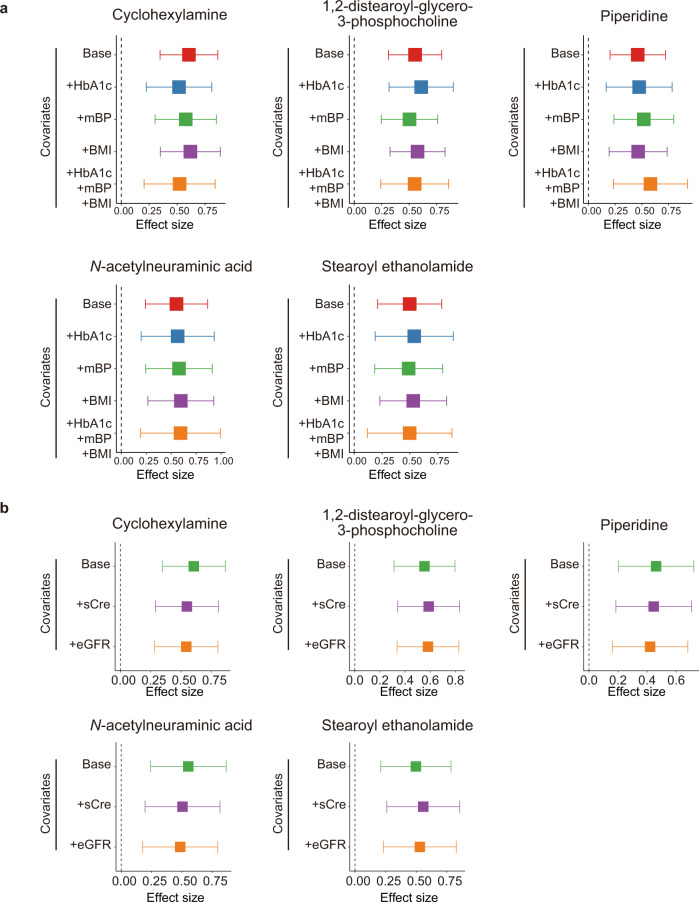
Forest plots from the results of the sub-analyses for the metabolites with significant association to the complications of T2D. The effect sizes of the sub-analyses for the metabolites with significant association to DR + DKD. The sub-analyses are performed for the **a** risk factors of the complications and **b** the markers of the renal function by logistic regression. The boxes indicate the point estimates, and the error bars indicate the 95% confidence interval. Number of the samples used for sub-analyses are following; Base, *N*_DR + DKD_ = 141, *N*_control_ = 159; +HbA1c, *N*_DR + DKD_ = 109, *N*_control_ = 112; +mBP, *N*_DR + DKD_ = 131, *N*_control_ = 132; +BMI, *N*_DR + DKD_ = 135, *N*_control_ = 147; +HbA1c+mBP+BMI, *N*_DR + DKD_ = 98, *N*_control_ = 93; +sCre and +eGFR, *N*_DR + DKD_ = 139, *N*_control_ = 157. BMI, body mass index; DKD, diabetic kidney disease; DR diabetic retinopathy; eGFR, estimated glomerular filtration rate; HbA1c, hemoglobin A1c; mBP, mean blood pressure; sCre, serum creatinine; T2D, type 2 diabetes.

To determine whether the identified associations were mediated by established risk factors for the complications of T2D such as HbA1c, mBP, and BMI, we performed sub-analyses with adjustment of HbA1c, mBP, and BMI. Since HbA1c, mBP, and BMI were not registered in the BBJ database for some participants, those with missing values were excluded from sub-analyses. As for all the five metabolites which had significant associations with the complications of T2D, effect sizes were stably consistent among the sub-analyses (Fig. 2a), suggesting that the associations were not confounded by either HbA1c, mBP, BMI alone or combination of HbA1c, mBP, and BMI.

We also evaluated whether the impaired renal function in those with the complications could affect the results. We performed sub-analyses with adjustment of sCre or eGFR and found that effect sizes were stably consistent among the sub-analyses (Fig. 2b). From these results, we concluded that our result was not confounded by the renal function, possibly because we recruited those with early-stage DKD.

**Fig. 3:**
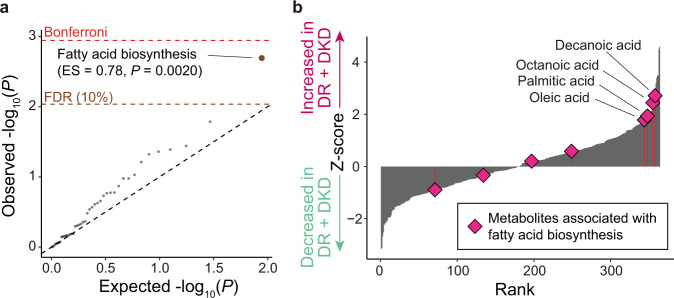
Results of the metabolite set enrichment analysis. **a** A quantile–quantile plot of the *p* values of pathways in MSEA based on the KEGG pathways. The *x*-axis indicates log-transformed expected *p* values. The *y*-axis indicates log-transformed observed *p* values. The diagonal dashed line represents *y* = *x*, which corresponds to the null hypothesis. The horizontal red dashed line indicates the Bonferroni-corrected threshold (*α* = 0.05), and the brown dashed line indicates the FDR threshold (FDR = 0.10) calculated with the Benjamini-Hochberg method. Pathways with *p* values less than the FDR thresholds are plotted as brown dots. **b** Bar-plot of the Z-scores in case–control association tests. The *x*-axis indicates the rank of the metabolites according to their Z-score. the *y*-axis indicates the Z-scores of the metabolites. Rhombus indicates the metabolites which are annotated as fatty acid biosynthesis-related metabolites. The number of samples used for the analysis is *N*_DR + DKD_ = 141 and *N*_control_ = 159. DKD, diabetic kidney disease; DR diabetic retinopathy; ES, enrichment score; FDR, false discovery rate; KEGG, Kyoto Encyclopedia of Genes and Genomes; MSEA, metabolite set enrichment analysis.

We performed pathway enrichment analysis using the result of the association tests for individual metabolites. We identified a significant association for the KEGG pathway of fatty acid biosynthesis (enrichment score = 0.78, *P* = 0.0020; Fig. 3a). Among the metabolites included in the fatty acid biosynthesis pathway, decanoic acid, octanoic acid, palmitic acid, and oleic acid drove the association (Fig. 3b). Among the KEGG pathways included in our MSEA, another fatty acid-related pathway, biosynthesis of unsaturated fatty acids was nominally enriched (enrichment score = 0.55, *P* = 0.036). Other fatty acid-related pathways such as beta-oxidation and fatty acid degradation were not included in the evaluated set of pathways.

## Discussion

In this study, we measured the serum metabolite abundance of Japanese persons with T2D with or without renal and retinal complications. Our analyses identified five metabolites that increased in persons with T2D with complications compared to those without complications. Among the complications-associated metabolites, *N*-acetylneuraminic acid was the major sialic acid in humans, and sialic acid was reported to be associated with DR^18^ and DKD^17^. Therefore, our large-scale multi-center analysis successfully identified the complication-associated metabolite, which was replicated in independent previous studies. Although we utilized an established commercial metabolomics service, it performed normalization and QC based on the single internal standard rather than using pooled QC samples, and it could be a potential limitation of this study. Future studies in other cohorts are warranted to further support the associations identified in our study. Even when HbA1c, mBP, and BMI were adjusted, the result of our association tests was stably consistent, suggesting that the identified associations were independent of the previously established risk factors for the complications of T2D.

*N*-acetylneuraminic acid was associated with myocardial infarction^19^. Given that there were associations between cardiovascular complications and retinal^8,20^ or renal^7^ complication of persons with T2D, *N*-acetylneuraminic acid could contribute to the shared etiology among the cardiovascular, retinal, and renal complications of persons with T2D. Myocardial infarction model rats which were knocked down Neuraminidase1, an *N*-acetylneuraminic acid-producing enzyme, showed reduced myocardial damage compared to the control rats due to the reduced accumulation of the inflammatory cells to the ischemic site^21^. The contribution of the *N*-acetylneuraminic acid to the pathogenesis of myocardial infarction via inflammation suggested that *N*-acetylneuraminic acid could contribute to the pathogenesis of DR and DKD via the inflammation. *N*-acetylneuraminic acid was reported to bind to the RhoA and Cdc42 and activate Rho/Rho-associated coiled-coil containing protein kinase (ROCK) signaling pathway which was involved in various biological processes including inflammation^21,22^. In persons with diabetes, activation of ROCK was reported for kidney^22^, retinal vessels, and retinal pigment epithelium^23^, suggesting that ROCK activation induced by *N*-acetylneuraminic acid could be involved in the pathogenesis of the DR and DKD.

Additionally, piperidine was detected as a complication-associated metabolite. Piperidine was previously reported to have pharmacological activities to regulate blood flow and vascular resistance via the muscarinic receptor and sympathetic nervous system^24^, suggesting its potential contribution to the vascular-associated complications of T2D. Piperidine was also reported as one of the metabolites which could be utilized for predicting retinopathy of prematurity (ROP)^25^. Since the microvasculature of the retina is affected in ROP, piperidine might contribute to the complications of T2D via microvascular-associated pathology.

Our MSEA revealed the enrichment of the fatty acid biosynthesis-related metabolites in persons with T2D with complications. In T2D, increased insulin resistance leads to compensatory hyperinsulinemia, which causes inflammation^26^. Since inflammation promotes the fatty acid biosynthesis in the liver^27^, our result could reflect the severer inflammation due to the increased insulin resistance in persons with T2D with the complications.

In conclusion, our metabolomic analysis identified the metabolomic features of persons with T2D with complications. These findings will contribute to revealing the etiology of DR and DKD. Additionally, T2D complications-associated metabolites identified in our study will be potential biomarkers for the early identification of persons with T2D with critical complications.

## Supplementary information

Supplementary Information-New

Description of Additional Supplementary Files

Supplementary Data 1

## Data Availability

The metabolite abundance data and summary statistics of the logistic regression analysis are available in NBDC Human Database (http://humandbs.biosciencedbc.jp/) with the accession number of hum0372 (note that metabolite abundance data provided by HMT was not raw spectrum data but table data). Source data for the main figures in this manuscript are provided as Supplementary Data 1.

## Code availability

Codes used for the analysis are available in Zenodo repository (10.5281/zenodo.7049152)^28^.
