# Supplementary material for: Identification of serum metabolome signatures associated with retinal and renal complications of type 2 diabetes
Source: Commun Med (Lond). 2023 Jan 9;3:5. doi: 10.1038/s43856-022-00231-3 (PMC9829655; doi:10.1038/s43856-022-00231-3)
Supplement: Supplementary file 1 — Supplementary Information-New [file 43856_2022_231_MOESM1_ESM.pdf]

Supplementary information

Identification of serum metabolome signatures associated with retinal and renal complications of type 2 diabetes

Yoshihiko Tomofuji, Ken Suzuki, Toshihiro Kishikawa, Nobuhiro Shojima, Jun Hosoe, Kyoko Inagaki, Sunao Matsubayashi, Hisamitsu Ishihara, Hirotaka Watada, Yasushi Ishigaki, The BioBank Japan Project, Hidenori Inohara, Yoshinori Murakami, Koichi Matsuda, Yukinori Okada, Toshimasa Yamauchi, Takashi Kadowaki

Table of contents

■Supplementary Figures.. 1

1. Results of the linear regression analysis to obtain the covariates-adjusted metabolite abundances 1
2. Raw abundances of the metabolites associated with the complications of the T2D 3

■Supplementary table 4

1. Wilcoxon rank sum test for the metabolites associated with the complications of T2D 4

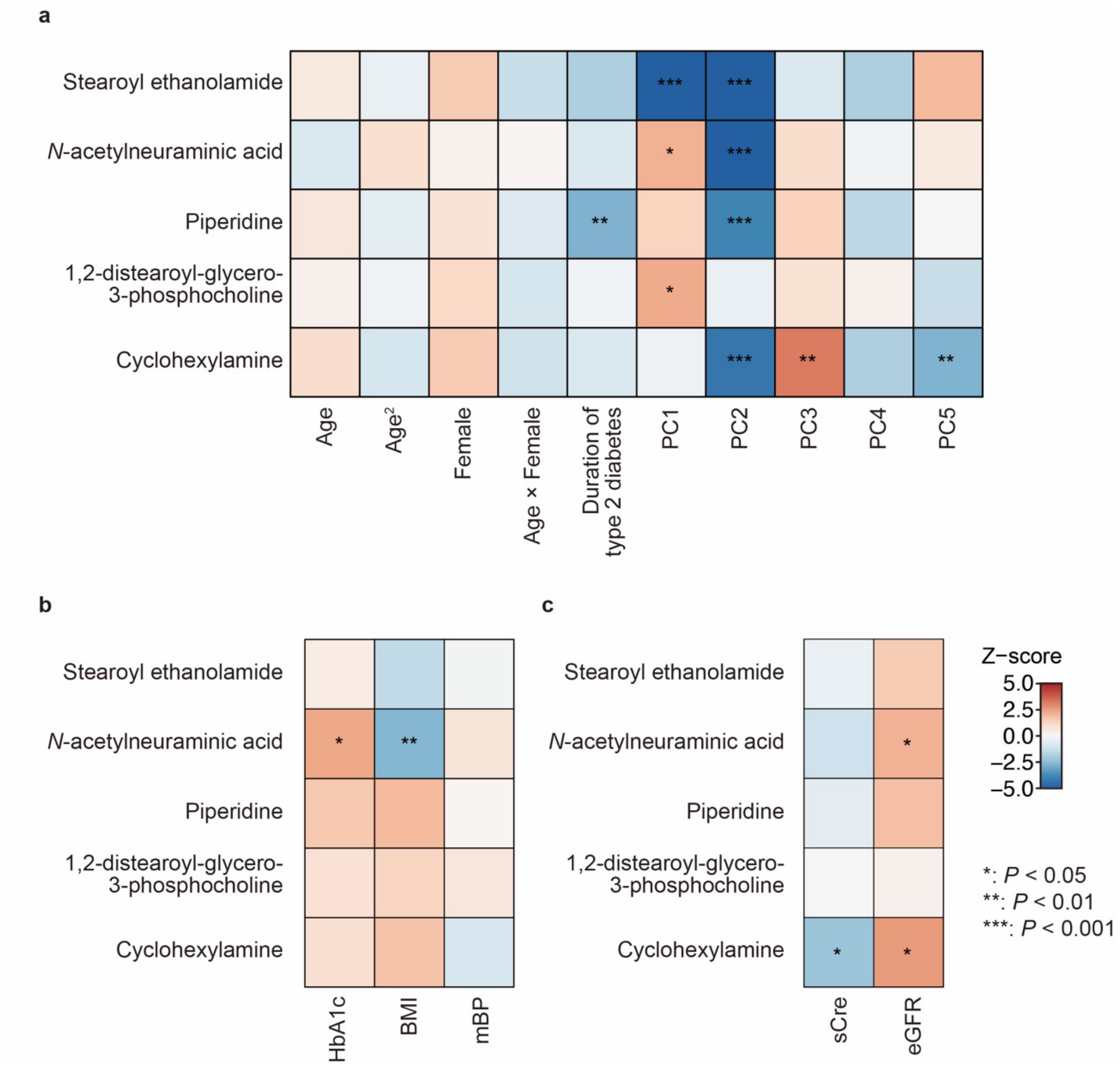

Supplementary Fig. 1 | Results of the linear regression analysis to obtain the covariates-adjusted metabolite abundances.

Heatmaps represent the Z-score of the covariates in the linear regression to obtain the covariate-adjusted metabolite abundances. (a), (b), and (c) correspond to **Fig. 1**, **Fig. 2a**, and **Fig. 2b**, respectively. The x-axis indicates the covariates and the y-axis indicates the metabolites. The color of the tile indicates the Z-score (effect size / standard error) in the linear regression. The asterisk indicates the significance of Wald's tests. Number of samples used for the analyses are following; **a**, $N_{\mathrm{DR+DKD}}$ = 141 and N_{control} = 159 for all the covariates; **b**, $N_{\mathrm{DR+DKD}}$ = 109 and N_{control} = 112 for HbA1c, $N_{\mathrm{DR+DKD}}$ = 131 and N_{control} = 132 for mBP, and $N_{\mathrm{DR+DKD}}$ = 135 and N_{control} = 147 for BMI; **c**, $N_{\mathrm{DR+DKD}}$ = 139, N_{control} = 157 for sCre and eGFR.

BMI, body mass index; DKD, diabetic kidney disease; DR diabetic retinopathy; eGFR, estimated glomerular filtration rate; HbA1c, hemoglobin A1c; mBP, mean blood pressure; PC, principal component; sCre, serum creatinine.

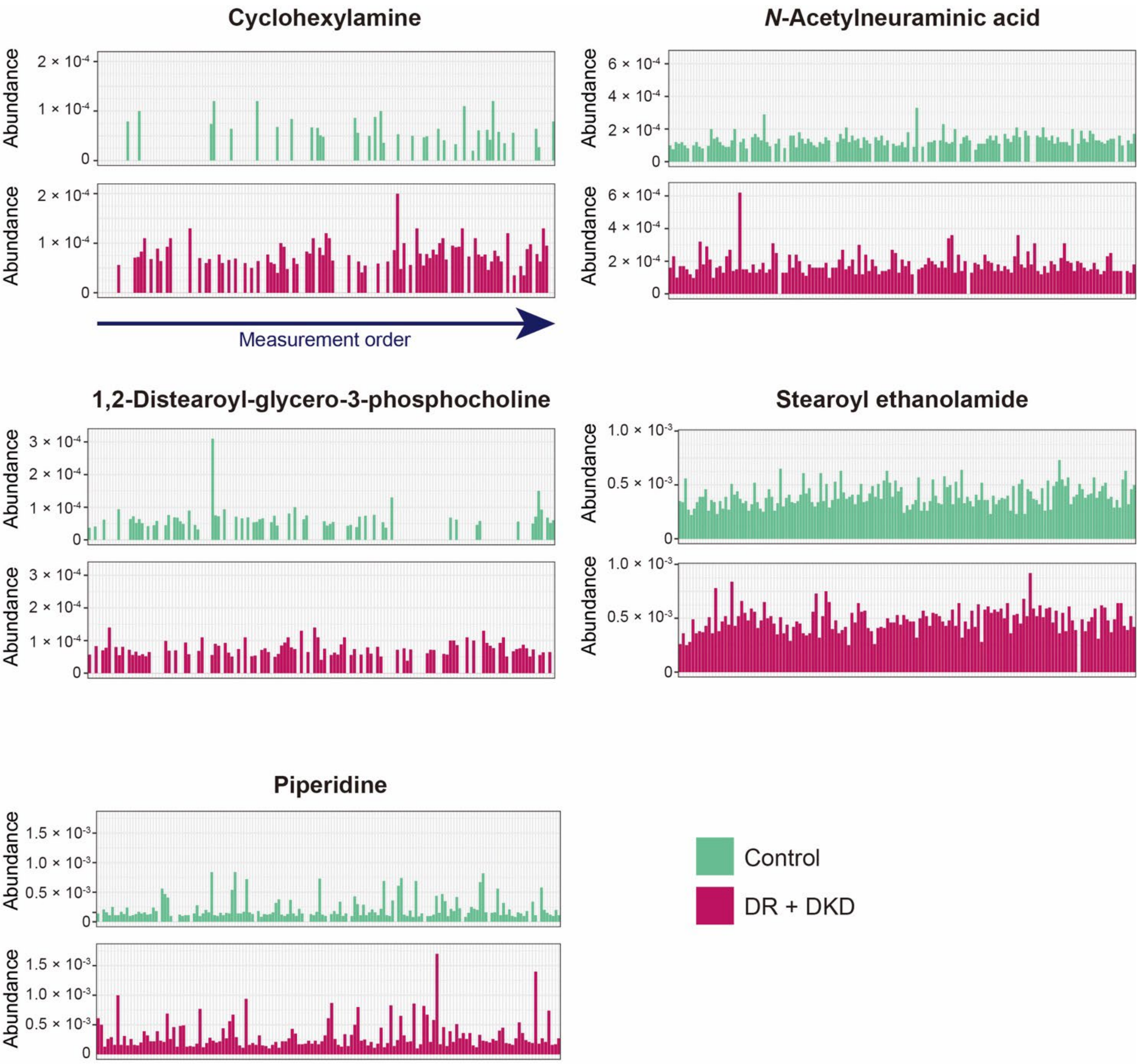

Supplementary Fig. 2 | Raw abundances of the metabolites associated with the complications of the T2D.

Bar plots represent the raw abundances of the metabolites associated with the complications of T2D. The x-axis indicates the order of the measurements and the y-axis indicates the raw abundances of the metabolites. The number of samples used for the analysis is $N_{DR + DKD}$ = 141 and $N_{control}$ = 159. DKD, diabetic kidney disease; DR diabetic retinopathy; T2D, type 2 diabetes.

Supplementary Table 1 | Wilcoxon rank sum test for the metabolites associated with the complications of T2D.

Metabolite	Control				DR + DKD				*P*
	Median	1st Q.	3rd Q.	*N*	Median	1st Q.	3rd Q.	*N*	
Cyclohexylamine	0.0	0.0	0.0	159	5.6×10^{-5}	0.0	7.7×10^{-5}	141	6.1×10^{-12}
1,2-Distearoyl-glycero-3-phosphocholine	0.0	0.0	5.5×10^{-5}	159	5.7×10^{-5}	0.0	7.6×10^{-5}	141	7.1×10^{-8}
Piperidine	1.4×10^{-4}	1.1×10^{-4}	2.2×10^{-4}	159	2.3×10^{-4}	1.7×10^{-4}	3.6×10^{-4}	141	1.4×10^{-13}
N-Acetylneuraminic acid	1.2×10^{-4}	9.5×10^{-5}	1.5×10^{-4}	159	1.6×10^{-4}	1.4×10^{-4}	2.0×10^{-4}	141	1.8×10^{-19}
Stearoyl ethanolamide	3.8×10^{-4}	3.3×10^{-4}	4.8×10^{-4}	159	4.8×10^{-4}	4.1×10^{-4}	5.6×10^{-4}	141	3.7×10^{-10}

1st Q., first quantile; 3rd Q., third quantile; DKD, diabetic kidney disease; DR diabetic retinopathy; T2D, type 2 diabetes.
