# Supplementary material for: Identification of serum metabolome signatures associated with retinal and renal complications of type 2 diabetes
Source: Commun Med (Lond). 2023 Jan 9;3:5. doi: 10.1038/s43856-022-00231-3 (PMC9829655; doi:10.1038/s43856-022-00231-3)
Supplement: Supplementary file 2 — Description of Additional Supplementary Files [file 43856_2022_231_MOESM2_ESM.pdf]

Description of Additional Supplementary Files

File Name: Supplementary Data 1

Description: Source data for the main figures
